# Non union of scaphoid fracture in a cricketer – possibility of a stress fracture: a case report

**DOI:** 10.1186/1752-1947-1-37

**Published:** 2007-06-29

**Authors:** Ulfin Rethnam, Rajam SU Yesupalan, Thirumoolanathan M Kumar

**Affiliations:** 1 Department of Orthopaedics, Glan Clwyd Hospital, Rhyl, UK

## Abstract

Scaphoid stress fractures are rare and can be a cause of wrist pain in sportspersons. All the cases reported in the literature have been sportspersons. Missing a scaphoid stress fracture could lead to non-union of the scaphoid and early degenerative arthritis of the radio-carpal joint. This can cause chronic wrist pain and can reduce the career span of a sportsperson. We report a case of non union of a scaphoid fracture in a cricketer possibly secondary to a stress fracture.

## Background

A 38 year old cricketer presented with a painful right wrist after being knocked by a cricket ball on the volar aspect. There was no history of significant injury to the wrist in the past although he did complain of occasional pain in the wrist for 2 years prior to his presentation. He was a keen cricketer and played at least twice a week for about 10 years.

## Case presentation

**Figure 1:**
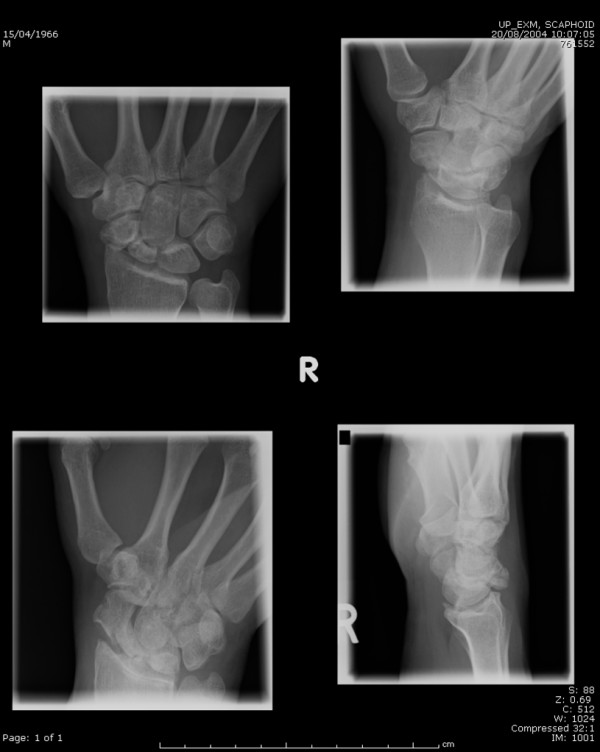
Scaphoid views showing non union of the scaphoid with associated radiocarpal arthritis.

On examination there was minimal swelling over the volar radial aspect of the wrist. He was tender over the radial styloid and the scaphoid. There was also a restriction of volar and dorsiflexion of the wrist due to pain. Radiographs revealed scaphoid fracture non-union and secondary osteoarthritic changes of the distal radius and carpal bones suggesting an old injury [Figure 1]. The wrist was placed in a removable splint for pain relief and the patient followed up. As the wrist already showed evidence of osteoarthritis, no fixation and bone grafting of the scaphoid non-union was done. The patient had mild wrist pain but no restriction of wrist movements at his last follow-up (1 year) although he has not been able to play cricket. He has been kept under regular review to detect and treat worsening arthritis of the wrist.

## Discussion

Stress fracture of the scaphoid is a rare yet important condition which if not detected can lead to non-union with secondary osteoarthritis of the wrist, which results in lifelong disability. There are only few cases reported in the literature. These have been previously reported in gymnasts[1], shotputter [2], and a badminton player[3]. In a review of literature a stress fracture of the scaphoid has not been reported in a cricketer. The mechanism of a stress fracture of the scaphoid has been postulated as repeated dorsiflexion of the wrist [2]. All these cases went on to heal after a period of immobilisation.

A cause for the scaphoid fracture could not be identified in this case as there was no history of significant injury to the wrist. Since the patient was a regular cricketer and had occasional pain in the wrist for 2 years, this could have been a stress fracture caused due to playing cricket. In this case repeated dorsiflexion of the wrist while batting or in bowling prior to the release of the ball could have resulted in the stress fracture. As the wrist pain was ignored he developed a non union of the scaphoid fracture with subsequent degenerative arthritis of the radiocarpal joint.

Young people are more prone to wrist injuries due to their involvement in sports. As non union of a scaphoid fracture can cause degenerative arthritis of the radio-carpal articulation any sports person presenting with wrist pain should be investigated for a scaphoid fracture.

## Conclusion

Scaphoid stress fractures have only been reported in sportspersons. These can cause degenerative arthritis of the radio-carpal articulation secondary to non-union if not detected and treated. The disability these can cause to young sportspersons cannot be understated. This report highlights this rare but important fracture.

## Competing interests

The author(s) declare that they have no competing interests.

## Authors' contributions

UR was involved in collecting patient details, reviewing the literature and drafted the manuscript as the main author. RSUY was involved in reviewing the literature and proof reading of the manuscript. RSUY has approved the final manuscript. TMK, the Senior author was responsible for proof reading the final draft.
